# The effect of participation in support groups on retirement syndrome in older adults

**DOI:** 10.1186/s12877-024-04923-4

**Published:** 2024-04-12

**Authors:** Sakineh Qorbani, Zahra Amrollah Majdabadi, Nasrin Nikpeyma, Shima Haghani, Sahar Keyvanloo Shahrestanaki, Sarieh Poortaghi

**Affiliations:** 1 Department of Community Health and Geriatric Nursing, School of Nursing and Midwifery, Tehran University of Medical Sciences, Tehran, Iran; 2 Department of Community Health and Geriatric Nursing, School of Nursing and Midwifery, Tehran University of Medical Sciences, Tehran, Iran; 3 Biostatistics Nursing Care Research Center, Iran University of Medical Sciences, Tehran, Iran

**Keywords:** Older adults, Retirement syndrome, Support groups

## Abstract

**Background:**

Retirement and aging are phenomena that often occur simultaneously and lead to various physical and psychological changes in older adults. Retirement syndrome consists of symptoms such as feelings of emptiness, loneliness, uselessness, lack of clear understanding of future conditions and dissatisfaction with one’s performance after retirement. This phenomenon requires interventions to adapt to these changes. Considering the supportive role of nurses, the formation of support groups as an effective intervention in adapting to transitional stages is emphasized.

**Aims:**

This study aims to investigate the effect of older adults’ participation in support groups on retirement syndrome.

**Methods:**

This Quasi-experimental study recruited a total of 80 retired older adults meeting the inclusion criteria from three Retirement Associations (Retirement centers for social security retirees are among the institutions that have been set up by the government and this organization to provide face-to-face and offline services to social security retirees, as well as providing some facilities to this segment of the society). in Iran, Research samples were randomly assigned to two intervention and control groups. The demographic questionnaire and retirement syndrome questionnaire were completed by both groups at the beginning of the study. Then, four support group sessions lasting 60 to 90 min were held twice a week for the support group, and eight weeks after the end of the intervention, the questionnaires were completed for both groups. The data were analyzed using statistical tests in SPSS version 16. The significance level was set at *p* < 0.05 for all tests.

**Results:**

The results of the covariance analysis showed that after the intervention, the feelings of helplessness and failure (*p* < 0.001), feelings of older and idleness (*p* = 0.027), and feelings of confusion and conflict (*p* = 0.002) were significantly less in the support group compared to the control group. In addition, the Feeling of trying and new direction (*p* < 0.001) was higher after the intervention. The paired t-test results showed that in the support group, the feelings of helplessness and failure (*p* < 0.001), feelings of older and idleness (*p* = 0.004), and feelings of confusion and conflict (*p* < 0.001) significantly decreased after the intervention compared to before it, while the feelings of trying and new direction (*p* = 0.004) significantly increased. Therefore, the results showed that after the intervention, there was a statistically significant difference between the two groups in all components of the retirement syndrome.

**Conclusion:**

The results of this study show that participation of retired older adults in support groups can significantly improve all components of retirement syndrome, leading to an improvement in their quality of life and satisfaction.

## Background

In modern times, various social, economic, health, and medical progressions have contributed to a decline in mortality rates and a significant rise in the life expectancy of people. Consequently, older adult population and retirees have witnessed a notable surge in the demographic makeup of numerous countries worldwide [1]. The global number of retirees is continuously on the rise, and projections indicate that it is likely to double in most countries by the year 2050 [2]. Notably, Iran has also observed a growth in the number of retirees, as evidenced by available statistical data [3].

Retirement represents a significant milestone in life, signifying a transition to a new stage with distinct roles, expectations, challenges, and opportunities [4]. Retirement and old age often coincide, leading to substantial changes in physical, psychological, occupational, and relational aspects that shape the lives of older adults [5]; [6]. The combination of symptoms experienced upon retirement is commonly referred to as retirement syndrome, encompassing feelings of helplessness, failure, aging, stagnation, the pursuit of a new direction, confusion, and conflict [7]. Studies have established a correlation between retirement syndrome’s components and general health issues such as physical ailments, anxiety, insomnia, social dysfunction, and depression among older adults [8]. This syndrome is characterized by its stressful, multifaceted nature, accompanied by various challenges that, over time, create deficiencies in the lives of retirees and impose significant costs on individuals and society [9]. Adapting to retirement as a new phase that brings about fundamental changes is crucial. However, many retirees struggle to adapt due to specific physical and psychological conditions, economic challenges, and age-related illnesses [10, 11]. Retirement alters numerous aspects of daily life, impacting retirement adaptation and various dimensions, including satisfaction, stress, inertia, hope, regret, anger, and depression among older adults [12, 13]. Given the range of emotional and psychological challenges, such as depression, stress, anxiety, declining physical health, and loss of social status, addressing retirement changes requires fundamental interventions and specialized attention [14].

One of the effective interventions for addressing retirement challenges is participating in support groups. Support groups are educational methods that can be tailored and implemented for diverse populations based on their specific needs. These groups can be valuable in offering health interventions encompassing physical, psychological, and spiritual aspects, while also considering the cultural context of the society [15]. Support groups have a long-standing history, and their core foundation lies in facilitating the sharing of experiences and providing feedback among group members. By engaging in these groups, individuals have the opportunity to be heard, and understood, gain self-awareness, and alter their perspectives with the assistance and support of a trained facilitator. The essence of these groups rests on the principle that supportive interactions with others who have undergone similar challenges can lead to the development of increased adaptive skills and an enhanced quality of life for individuals [16].

In the Nursing Interventions Classification (NIC), support groups are recognized as an intervention aimed at helping individuals establish adaptability during transitional stages [17]. Within these groups, nurses play a crucial role by facilitating group discussions, providing accurate scientific information, maintaining group dynamics (ensuring equal participation and problem-solving), encouraging effective communication among members, and offering feedback while adhering to group rules [18]. Nurses can play an important role in helping the elderly in social empowerment and successful adaptation to the environment because nursing personnel are considered the main source of supportive and non-supportive behaviors related to emotional and practical support in facing the challenges of old age. The elderly population in Iran, particularly during retirement, encounters numerous challenges. Many older individuals grapple with financial insecurity stemming from inadequate retirement benefits and a lack of social support systems [19]. In Iran, geriatric nurses have the opportunity to work in various settings, and part of their responsibility involves establishing support groups, providing guidance, and assisting the elderly in adapting to the challenges of aging, especially at the community level [18].

Although numerous studies have highlighted the positive impact of participating in support groups [20], there has been no specific investigation into the effect of support groups on retirement syndrome, particularly within the cultural context of Iran. Additionally, no dedicated support and care programs for retirees have been implemented in the country. Considering the increasing population of older adult retirees and the potential consequences on their physical, psychological, and social health, it becomes vital to focus on this stage due to its overlap with older adults phase, leading to potential compounded problems.

In light of the significance of support groups as an essential nursing intervention for enhancing adaptability during transitional stages, and the researcher’s role as a geriatric nurse with strong communication skills with the older adults, this study aims to explore the impact of older adults’ participation in support groups on retirement syndrome. The investigation seeks to shed light on the potential benefits of support group participation for retirees and contribute to improving their overall living conditions.

### Study objective

The present study was conducted to examine the effect of older adult’s participation in support groups on retirement syndrome.

## Methodology

The current study utilized a quasi-experimental research design and was conducted at Retirement Associations, including National, Tamin Ejtemai, and Alborz, located in the city of Savadkuh, Iran, during the period of 2020–2021.

### Sampling

The sampling process for the study involved two steps: initial sampling and random assignment of participants to the support group and control groups using block randomization.

For the initial sampling, participants were selected based on availability and public announcements. Eligible individuals were then randomly assigned to either the support group or control group using block randomization. The random sequence was generated using a computer program from the website https://www.randomizer.org, resulting in 20 four-member blocks. Each block was given a number, and all possible combinations of the four-member blocks were determined. For each block, two participants were allocated to the support group, and two to the control group, leading to six possible combinations.

To maintain the randomness of the sequence, 80 opaque envelopes were prepared to conceal the content. The randomly generated sequences were recorded on registration cards, which were then placed inside the corresponding envelopes in sequential order. The envelopes were numbered on the outer surface in the same order to preserve the random sequence. Subsequently, the envelopes were sealed and placed in a box in sequential order.

During participant registration, the envelopes were opened in order of eligibility, revealing the assigned group for each participant.

To determine the required sample size, the study considered a 95% confidence level, 80% power of the test, a precision of 0.05, and the estimated standard deviation from similar studies. The minimum sample size calculated for each group was 35 participants. Accounting for potential dropouts, the sample size in each group was increased to 40 participants.

$$ n=\frac{({{z}_{1-\raisebox{1ex}{$\alpha $}\!\left/ \!\raisebox{-1ex}{$2$}\right.}+{z}_{1-\beta })}^{2}\times ({s}_{1}^{2}+{s}_{2}^{2})}{{d}^{2}}$$

$$ n=\frac{({1.96+0.84)}^{2}\times (2\times {0.75}^{2})}{{0.5}^{2}}=35$$

The inclusion criteria for participants in the study were as follows: membership in Retirement Associations, age over 60 years, not being re-employed after retirement, no self-reported prominent cognitive disorders, such as Alzheimer’s, dementia, or delirium, ability to establish visual and verbal communication, a minimum of 6 months since retirement. The exclusion criteria for participants were: unwillingness to continue participation in the study, absence from two consecutive support group sessions, and onset of cognitive impairment during the study, and occurrence of a significant event during the study implementation.

### Data collection tools

The data collection tools utilized in this study consisted of two questionnaires: a demographic questionnaire and a retirement syndrome questionnaire. The researcher designed the demographic questionnaire, which collected information such as age, gender, education level, marital status, previous occupation, years of service, reason for retirement, and any associated illnesses. The retirement syndrome questionnaire consisted of four components and 40 items, which were designed and validated by Bozorgmehri et al. (2008). The four main components, based on the content of the items, were feelings of helplessness and failure (18 items), feelings of aging and uselessness (11 items), feelings of trying and new direction (6 items), and feelings of confusion and conflict (5 items). The response scale for this questionnaire was a five-point scale ranging from “never” to “always.” It should be noted that questions 1, 2, 4, 9, 24, 32, and 39 were reverse-scored (rated as “always” = 1 to “never” = 5). Furthermore, due to the reverse scoring and the presence of three negative components (feelings of helplessness and failure, feelings of aging and uselessness, and feelings of confusion and conflict) and one positive component (feelings of trying and new direction), it was not possible to calculate a total score for this questionnaire.

The reliability of the retirement syndrome questionnaire was evaluated using internal consistency and Cronbach’s alpha coefficients.

The overall Cronbach’s alpha coefficient for the entire questionnaire was calculated to be 0.714. These values indicate a moderate to good level of internal consistency for the questionnaire [21].

In this study, the reliability of the retirement syndrome questionnaire was further assessed using Cronbach’s alpha coefficient for a sample of 20 retired older adult individuals.

The overall Cronbach’s alpha coefficient for the entire questionnaire was calculated to be 0.657. These values indicate a moderate level of internal consistency for the questionnaire when applied to this specific sample of retired older adult’s individuals.

### Intervention method and data analysis

After selecting eligible participants, the researchers provided them with detailed explanations about the research and obtained informed consent from those willing to participate. Subsequently, all participants completed the demographic questionnaire and the retirement syndrome questionnaire. Random allocation was then performed to assign individuals to either the control group (40 participants) or the support group, which was further divided into four groups of ten retired older adult individuals who met the inclusion criteria. Each support group had facilitated by a nurse serving as the group leader, and a successful peer who was an individual with similar characteristics, such as age, gender, occupation, socioeconomic status, and health status, with other group members. They are capable of sharing their experiences, strengths, and weaknesses, and they encourage individuals to adopt appropriate health behaviors through practical, emotional, and informational support.

Supportive group programs were developed for each of the four support groups, and each session lasted for 60 to 90 min, conducted twice a week based on the participants’ tolerance levels. These sessions took place in a facility under the municipality’s supervision in Savadkuh City, Iran.

During each session, the nurse initiated a discussion by presenting a predetermined indirect scenario, which had been validated by the research team and a psychologist. These scenarios depicted the experiences of four retired older adult individuals, each of whom had encountered one of the retirement syndrome components (helplessness and failure, aging and uselessness, trying and new direction, and confusion and conflict). Group members were then encouraged to share their own similar experiences and discuss the coping strategies they had employed. Participants engaged in discussions, exchanged opinions, provided emotional support to each other, and benefited from the successful peer’s experiences related to the topic under discussion. At the end of each session, the nurse summarized the discussed and resolved any doubts that arose for the elderly participants. The study highlights the importance of the nurse’s role in leading and guiding the support group sessions, as well as the interactions and discussions within the group. The meaning of support groups in this intervention was interventions designed by specialists (geriatric nurses) to provide emotional, psychological, and educational support for a group of people who have a common condition or problem. In these support groups, the experiences and feelings of people who had gone through similar situations were shared. In this study, the method of support groups under the guidance of an expert was used, which focused on the development of treatment goals. In these groups, an opportunity was provided for mutual support and education with common psychological and rehabilitation goals under the leadership and guidance of an expert. The main component of the support group was sharing experiences and providing feedback.

Eight weeks after the completion of the intervention sessions, the retirement syndrome questionnaire was administered again to the participants, and the results were compared with the initial scores. Throughout the face-to-face sessions, the researcher provided their contact number to address any potential issues or concerns raised by the research participants.

The topics determined in each session based on the components of retirement syndrome based on indirect scenarios included the following:

The first session: a short introduction, a presentation of an indirect scenario with the topic of helplessness and failure, The second session: presents an indirect scenario with the topic of feeling old and useless, The third session: presents an indirect scenario with the theme of feeling effort and new direction, The fourth session: presenting an indirect scenario with the topic of feeling confused and conflicted. Discussion and exchange of opinions and emotional support of the participants, summarization of the contents were carried out at the end of all sessions.

Data analysis was performed using SPSS version 16 software. Descriptive statistics (frequency, mean, standard deviation) and inferential statistics (independent t-test, paired t-test, chi-square test, Fisher’s exact test, analysis of covariance) were used for data analysis. The normality of quantitative variables was assessed using the Kolmogorov-Smirnov test, and their normality was confirmed. The significance level for all tests was set at *p* < 0.05.

## Result

Table 1 presents the demographic characteristics of the participants in both the intervention and control groups. The research sample consisted of 80 individuals, with mean ages of 67.50 ± 6.16 years in the support group and 65.25 ± 4.20 years in the control group. The majority of participants in both groups were male and married. The analysis of demographic characteristics indicated that both groups were homogeneous in terms of demographic variables.

**Table 1:** Frequency distribution of demographic characteristics of the participants in each group

		Intervention (*n* = 40)	Control (*n* = 40)	*p
Age M(SD)		67.50(6.16)	65.25 (4.2)	^†^0.061
Gender; n (%)	female	2(5%)	4(10%)	^&^0.675
	male	38(95%)	36(90%)	
Education n (%)	No educated	6(15%)	3(7.5%)	^&^0.609
	Under Diploma	27(67.5%)	29(72.5%)	
	Academic	7(17.5%)	8(20%)	
Marital status n (%)	Single	3(7.5)	2(5)	^&^0.999
	married	37(92.5%)	38(95%)	
Job n (%)	Employee	16(40)	20(50)	^††^0.369
	Free Job	24(60%)	20(50%)	
work experience(years); M(SD)		28.48(3.48)	27.45 (4.96)	^†^0.289
Reason for retirement n (%)	Finished work time	30(75%)	29(72.5)	^††^0.799
	else	10(25%)	11(27.5)	
Chronic disease; n (%)	yes	23(57.5)	20(50)	^††^0.501
	no	17(42.5)	20(50)	

*Significance level: *P* < 0.05 † Independent sample t-test ††Pearson’s chi-square test ^&^ Fisher Exact Test

Table 2 presents the results of the analysis of the retirement syndrome dimensions before and after the intervention in both groups. Before the intervention, there were statistically significant differences in the dimensions of feelings of helplessness and failure (*p* = 0.041) and feelings of confusion and conflict (*p* = 0.043) between the intervention and control groups. However, there were no statistically significant differences in the dimensions of feelings of aging and uselessness (*p* = 0.66) and feelings of trying and new direction (*p* = 0.56). After the intervention, the covariance analysis was used to adjust the pre-test scores. The results showed significant improvements in the support group in the dimensions of feelings of helplessness and failure (*p* < 0.001), feelings of confusion and conflict (*p* = 0.002), and feelings of aging and uselessness (*p* = 0.027). Additionally, there was a significant positive effect of the intervention on the dimension of feelings of trying and new direction (*p* = 0.001).

In the control group, there were no statistically significant differences in any of the dimensions of retirement syndrome before and after the intervention. However, in the support group, there were significant improvements in the dimensions of feelings of helplessness and failure (*p* = 0.001), feelings of confusion and conflict (*p* = 0.001), and feelings of aging and uselessness (*p* = 0.04), and a significant increase in the dimension of feelings of trying and new direction (*p* = 0.004).

The effect sizes (η^2) for the changes in the retirement syndrome dimensions in the support group were moderate to large, indicating a significant impact of the intervention on improving the retirement syndrome components.

Overall, the results demonstrate that the intervention had a positive effect on reducing feelings of helplessness and failure, feelings of confusion and conflict, and feelings of aging and uselessness while increasing feelings of trying and new direction in the older adult participants. The control group, which did not receive the intervention, showed no significant changes in the retirement syndrome dimensions during the study period.

**Table 2:** Mean and standard deviation of the retirement syndrome in each Group

		Intervention (*n* = 40)	Control (*n* = 40)	†*p*
Feeling helplessness and failure M(SD)	Before	2.92 (0.47)	2.69 (0.50)	†0.041
	After	2.63 (0.35)	2.70 (0.43)	‡0.001 ${{\eta }}^{2}$=0.439
	††p	< 0.001	0.656	
Feeling older and idleness M(SD)	Before	3.00 (0.36)	2.96 (0.38)	†0.666
	After	2.91 (0.25)	2.97 (0.34)	‡0.027 ${{\eta }}^{2}$=0.062
	††p	0.04	0.628	
Feeling of trying and new direction M(SD)	Before	2.72 (0.80)	2.82 (0.78)	†0.56
	After	2.89 (0.64)	2.76 (0.69)	‡0.001 ${{\eta }}^{2}$=0.127
	††p	0.004	0.223	
Feeling of Conflict and confusion M(SD)	Before	2.51 (0.45)	2.25 (0.65)	†0.043
	After	2.26 (0.36)	2.25 (0.54)	‡0.002 ${{\eta }}^{2}$=0.113
	††p	< 0.001	0.907	

*Significance level: *P* < 0.05 † Independent sample t-test †† Paired T-test ‡ ANCOVA test with adjusting the baseline score

${{\eta }}^{2}$= partial eta-squared=Effect sizes: 0.01 = small; 0.06 = moderate; 0.14 = large

## Discussion

This study aimed to investigate the impact of participation in support groups on the components of retirement syndrome in older adult’s individuals. The results revealed that support groups were effective in improving retirement syndrome components. Retirement is a significant life event that brings about major changes in the lives of the older adults, including disruptions in physical and mental health, social relationships, and various emotional experiences like helplessness, aging concerns, and conflicting emotions [22]. The study demonstrated that support group participation positively influenced retirement syndrome components, leading to improvements in feelings of loss and failure, aging concerns, trying new directions, and confusion and conflict, thus contributing to the well-being of retirees.

A noteworthy finding was the reduction in feelings of helplessness and failure among retired individuals after participating in the support groups. Retirees often face feelings of helplessness and failure as they adapt to leaving their jobs and losing their social status [23]. Such feelings can lead to various problems, including depression, anxiety disorders, and social dysfunction, adding to the psychological burden on the elderly [24]. Our study aligns with prior research indicating that participation in support groups and access to psychological support resources can positively impact retirees’ adaptation to retirement and enhance their motivation, leading to decreased feelings of helplessness and failure [25].

The study also demonstrated the effectiveness of group support in reducing feelings of aging concerns and idleness among retired elderly individuals. Aging often brings physical decline and concerns about feeling idle, significantly affecting the mental and social well-being of the older adults [26]. These feelings play a crucial role in predicting physical health and quality of life in seniors [19]. The study findings emphasize the importance of developing coping mechanisms for aging that promote self-esteem, worthiness, and overall well-being of retirees [22]. Active participation in social activities and support groups can be instrumental in addressing such concerns and improving life satisfaction among retirees, as supported by previous studies [27].

Furthermore, the intervention led to a positive effect on the feeling of trying new directions among retired individuals. Retirement offers an opportunity for retirees to make changes in their routines, learn new skills, and explore new goals [28]. Engaging in such endeavors has been shown to positively impact mental health, reduce mortality, alleviate depression symptoms, and increase life satisfaction in the older adults [29]. Conversely, retirees who resist change may experience feelings of uselessness, emptiness, and heightened anxiety [30]. Planning for successful retirement, engaging in meaningful activities, and providing support and guidance to retirees have been highlighted as factors contributing to improved quality of life [31]. Therefore, the findings underscore the importance of supportive and educational interventions for retirees to cultivate a sense of trying new directions and adapt positively to retirement.

Finally, the intervention also showed positive effects on reducing feelings of conflict and confusion among retired individuals. The lack of social recognition and respect after retirement can contribute to conflicting emotions and resistance to the transition from work to retirement [8]. Combined with age-related physical challenges, this can lead to confusion and ambiguity in retirees [32]. Studies have highlighted the role of group and social support in fostering positive retirement experiences and reducing feelings of conflict and confusion following retirement [33]. The present study’s findings align with this research, indicating that group interventions can effectively address and mitigate feelings of conflict and confusion among retirees.

To be concluded, the study demonstrated the positive impact of participation in support groups on the components of retirement syndrome in older adults’ individuals. These interventions can lead to improvements in feelings of helplessness and failure, aging concerns, trying new directions, and conflict and confusion. Nurses and caregivers in geriatric care should consider incorporating group support interventions to enhance the well-being and adaptation of retirees, ultimately promoting a positive and fulfilling retirement experience.

## Conclusion

In conclusion, this study highlights the importance of social support for retirees, considering the significant psychological and social challenges associated with retirement and their impact on the health of the older adults. Social support can effectively improve the well-being and protect retirees from various psychological consequences of retirement. Geriatric nurses have a crucial role to play in facilitating a smooth transition to retirement by implementing group support interventions to foster positive attitudes and adaptation among retired individuals.

Future research in this area should aim to expand the sample size and prolong the intervention duration to gain a more comprehensive understanding of the effects of such interventions on the overall quality of life of retirees. The current findings can also contribute to healthcare policy-making, guiding geriatric and community health nursing in planning effective strategies for supporting retirees during this transitional stage. By focusing on social support and improving the retirement experience for older adult’s individuals, we can enhance their overall well-being and promote a healthier and happier aging population.

## Limitations

The use of self-reported questionnaires may introduce response biases and social desirability bias, as participants may provide answers they think are more socially acceptable rather than their true feelings or experiences. To mitigate this limitation, the researcher assured participants of confidentiality and emphasized the importance of providing honest and accurate responses.

Furthermore, the study’s follow-up period was relatively short (eight weeks after the intervention), which may not capture the long-term effects of participation in support groups. Future studies could extend the follow-up period to assess the sustainability of the intervention’s effects over time.

Despite these limitations, the study provides valuable insights into the potential benefits of support group interventions for retired older adults’ individuals. By addressing these limitations in future research, we can gain a deeper understanding of the effectiveness of such interventions and improve the well-being and quality of life of retirees.

## Data Availability

All data and additional data files in Persian are available from the corresponding author on reasonable request.
